# Comment on “Takayasu Arteritis and Giant Cell Arteritis: Results From a Cross‐Sectional Study of 96 Italian Patients”

**DOI:** 10.1002/iid3.70513

**Published:** 2026-09-28

**Authors:** Shubhendu Mohanty, Adarsh Jyoti Lakra, Hima Bindu Mantravadi, Prerna Uniyal, Dhanya Dedeepya

**Affiliations:** ^1^ Department of Medicine School of Medical Sciences and Research, Sharda University Greater Noida India; ^2^ Dr. D. Y. Patil Medical College, Hospital and Research Centre, Dr. D. Y. Patil Vidyapeeth (Deemed‐to‐be‐University), Pimpri Pune India; ^3^ Department of Microbiology, Malla Reddy Institute of Medical Sciences Malla Reddy Vishwavidyapeeth (Deemed to be University) Hyderabad India; ^4^ Faculty of Pharmaceutical Sciences Graphic Era Hill University Dehradun India; ^5^ Centre for Promotion of Research Graphic Era Deemed University Dehradun India; ^6^ Saveetha Medical College and Hospital, Saveetha Institute of Medical and Technical Sciences Saveetha University Chennai India

Dear Editor,

1

Prete and colleagues compare 59 patients with Takayasu arteritis and 37 with giant cell arteritis across three Italian centers, and conclude that the two are clinically distinct conditions requiring disease‐specific diagnostic and therapeutic approaches [1]. Assembling a well‐characterized cohort of two rare vasculitides, imaged to a common protocol and followed for a median of 5 years, is difficult and valuable work, and the descriptive account of vascular distribution and treatment is a useful addition. Our concern is with the inference drawn from it.

The study asks whether Takayasu arteritis and giant cell arteritis are distinct entities or manifestations of a single disease. That question cannot be answered by a design in which the two groups are defined using the features on which they are then compared. Patients were allocated by the 2022 ACR/EULAR classification criteria, and those criteria are built from precisely the variables the paper reports as differences [2, 3]. Age is the clearest case: the Takayasu criteria require onset at or below 60 years and the giant cell arteritis criteria require age 50 or above, so a median age of 33 against 76 with *p* < 0.001 restates the entry rule rather than discovering anything. Vascular distribution is the more consequential case, because the methods say explicitly that the differentiation of cranial giant cell arteritis from the large‐vessel form and from Takayasu arteritis was based on the angiographic assessment. Table 2 then compares angiographic vascular distribution between the groups and reports axillary, aortic arch, mesenteric and renal artery involvement as distinguishing findings. The classifier and the outcome are the same variable. Polymyalgia rheumatica, present in 37.8% of the giant cell arteritis group and 0% of the Takayasu group, sits in the same category, since it is a recognized component of the giant cell arteritis phenotype that informs the diagnosis.

This is not an argument that the two diseases are the same. It is an argument that a cohort assembled by classification criteria cannot adjudicate the question, because classification criteria are constructed to discriminate between the entities they name and were validated for that purpose. The features that could adjudicate it are the ones the criteria do not use, and the paper has several: erythrocyte sedimentation rate and C‐reactive protein, which did not differ (*p* = 0.722 and *p* = 0.448), the neurologic and pulmonary manifestations, which did not differ, and the long‐term remission rate, which the abstract reports as not significantly different. A version of this analysis restricted to criteria‐independent variables would be a genuine test, and it would be a more interesting paper.

Second, the conclusion rests on counting significant differences across roughly fifty comparisons made at a threshold of 0.05 with no adjustment. Tables 1, 2 and 3 together report about thirty, eleven and ten tests respectively, and several of the differences carried into the conclusion are marginal: dermatologic involvement at *p* = 0.04, chronic liver disease at *p* = 0.02, axillary artery involvement at *p* = 0.02, and low‐dose glucocorticoid use at *p* = 0.04. At this number of tests, two or three findings at that level are expected by chance alone [4]. Fisher's exact test, which the authors chose appropriately for the sparse cells, addresses the validity of each individual test but does nothing about their number. We would separate a small number of prespecified comparisons from the remainder, and present the rest as exploratory.

Third, the treatment comparison is confounded by the referral pattern the authors themselves describe. The limitations note that one of the three centers is a regional tertiary referral center for Takayasu arteritis, and offer this as an explanation for the imbalance in group sizes. The consequence goes further than group size. A referral center concentrates severe and refractory disease, so the Takayasu group is enriched for exactly the patients who receive high‐dose glucocorticoids, combination immunosuppression and vascular intervention. The paper reports all three as differences between the diseases: high‐dose glucocorticoids in 59.3% against 18.9%, glucocorticoids plus immunosuppressants in 86.4% against 51.4%, and interventional procedures in 25.4% against 5.4%. These are differences between two differently recruited populations at least as much as between two diseases, and the conclusion that the conditions differ in their response to therapy does not follow from them.

Finally, several figures should be reconciled at proof. The abstract gives the time from symptom onset to diagnosis as *p* = 0.025 while Table 1 and the results text give *p* = 0.029. The caption to Table 3 describes a cohort of 84 patients, where the study comprises 96. The subclavian artery row of Table 2 gives the giant cell arteritis proportion as 35140, presumably 35.14%. And the proportion followed for at least 60 months appears as 72% in the abstract and discussion but 75% in the methods.

None of this diminishes the cohort. Our argument is narrower than the paper's conclusion and, we think, more defensible from these data: the two groups differ on the variables used to constitute them, do not differ on several that were not, and were drawn from centers with different case mixes. A comparison restricted to criteria‐independent features, with a prespecified analysis plan, would let this cohort speak to the question it raises.

## Author Contributions

**Shubhendu Mohanty:** writing – review and editing, writing – original draft. **Adarsh Jyoti Lakra:** writing – review and editing, writing – original draft. **Hima Bindu Mantravadi:** writing – review and editing, writing – original draft. **Prerna Uniyal:** writing – original draft, writing – review and editing. **Dhanya Dedeepya:** writing – original draft, writing – review and editing.

## Funding

The authors have nothing to report.

## Ethics Statement

Ethics approval was not required because this correspondence did not involve new research with human participants, animals, or identifiable personal data.

## Conflicts of Interest

The authors declare no conflicts of interest.

## Data Availability

Data sharing is not applicable because no new datasets were generated or analysed for this correspondence.
